# The value of immature granulocyte percentage united with D-Dimer in the evaluation of severe pancreatitis and its prognosis

**DOI:** 10.1016/j.clinsp.2024.100446

**Published:** 2024-07-14

**Authors:** Tian-Tian Xu, Si-Bing Chen

**Affiliations:** a Department of Critical Care Medicine, Shengjing Hospital, China Medical University, Shenyang City, Liaoning Province, China; b Department of Rehabilitation Medicine, Shengjing Hospital, China Medical University, Shenyang City, Liaoning Province, China

**Keywords:** Immature Granulocyte Percentage, D-dimer, Severe Pancreatitis, Prognosis, Evaluation Value

## Abstract

**Objective:**

The study aimed at analyzing the serum expression of Immature Granulocyte percentage (IG %) and D-Dimer (D-D) in patients with severe pancreatitis and exploring their clinical diagnostic value.

**Methods:**

Eighty-four cases with severe pancreatitis received in Shengjing Hospital, China Medical University from July 2020 to July 2023 were regarded as the study group and conducted for retrospective analysis. They were divided into a survival group (n = 62) and a death group (n = 22) based on the prognosis. Another 80 patients diagnosed with mild and moderate pancreatitis were selected as the control group. Serum IG % and D-D levels of all subjects were analyzed and the value of IG % and D-D in the evaluation of severe pancreatitis and its prognosis was conducted by Receiver Operating Characteristic (ROC) curve.

**Results:**

The IG % and D-D levels in the study group were markedly higher than the control group (p < 0.05). The IG % and D-D level in the death group were observably higher than the survival group (p < 0.05). The Area Under the Curve (AUC) of IG % and D-D combined assessment for severe pancreatitis was 0.963, and the sensitivity and specificity were 98.75 %, 82.14 %, respectively. The AUC of IG % and D-D combined assessment for prognosis of severe pancreatitis was 0.814 with a sensitivity of 79.03 % and a specificity of 77.27 %. The efficiency of joint evaluation of the two indicators is superior to the individual evaluation.

**Conclusion:**

Serum IG % and D-D are highly expressed in patients with severe pancreatitis, which has important clinical value for the evaluation of severe pancreatitis and its prognosis.

## Highlights

• The serum levels of IG % and D-D in the study and control group.
• The serum levels of IG % and D-D in the survival group and death group.
• Diagnostic value of IG % and D-D in severe pancreatitis.
• Assessment value of IG % and D-D in prognosis of severe pancreatitis patients.

## Introduction

In recent years, the incidence of acute pancreatitis has been increasing year by year.^1^ According to relevant studies, the incidence of severe acute pancreatitis is as high as 10 %∼20 %.^2^ Severe acute pancreatitis usually has a rapid onset, rapid disease progression, and a critical condition, and is prone to secondary infection, peritonitis, shock, and even multiple organ dysfunction, eventually leading to death.^3^^,^^4^ Therefore, the early diagnosis and prognosis evaluation of severe pancreatitis are highly valued in clinics.

At present, there are many clinical indicators and criteria for evaluating severe pancreatitis, but each evaluation indicator and system has its own advantages and disadvantages. For example, although the Bedside Index for Severity in Acute Pancreatitis (BISAP) score can effectively evaluate the severity and prognosis of acute pancreatitis, it does not show obvious superiority compared with the single predictor.^5^ Computed Tomography Severity Index (CTSI) score can reflect the disease condition and prognosis to a certain extent, but it has strict requirements on imaging equipment and the result interpretation is subjective to a certain extent.^6^ Therefore, it is of great significance to explore higher-quality indicators for the early diagnosis and prognosis assessment of this disease. Previous studies have shown that acute pancreatitis is a process mediated by a series of inflammatory responses, so it is believed that the disease progression may be closely related to inflammatory responses.^7^^,^^8^ Immature Granulocyte Percentage (IG %) is a newly discovered inflammatory marker that can be rapidly determined by routine serum examination. This index can accurately reflect the inflammation of the body and has important meaning in early diagnosis and prognosis evaluation of acute and critical diseases.^9^^,^^10^ D-Dimer (D-D) as an important indicator inflecting fibrinolytic activity, is significantly higher in patients with generalized inflammatory syndrome and has a definite value in assessing the disease status and prognosis of critically ill patients.^11^^,^^12^

Currently, IG % and D-D have shown some clinical value in the evaluation of severe pancreatitis, but they are usually combined with other indicators in actual clinical evaluation to improve the accuracy of the evaluation. Therefore, this study explored the evaluation value of the combination detection of two indexes in the diagnosis and prognosis of severe pancreatitis.

## Research methods

### Ethical approval of research protocol

Ethical approval was obtained from the hospital review board and written informed consent was obtained from patients or their guardians before participating in this study.

### Patients

Patients with severe pancreatitis received in Shengjing Hospital, China Medical University from July 2020 to July 2023 were enrolled in this study. Inclusion criteria: age 40∼75 years old; patient's clinical data were complete; patients have no other critical illness; all patients were followed up to obtain complete prognostic information. Exclusion criteria: patients with malignant tumors, cardiovascular and cerebrovascular diseases; patients who have dysfunction of liver and kidney; patients who died within 48 hours of admission; patients who used anti-inflammatory drugs within one month prior to admission. Based on these criteria, 84 patients were selected as study groups, and 80 patients diagnosed with mild and moderate pancreatitis during the same period were selected as the control group.

### Prognosis assessment

Taking admission diagnosis of severe pancreatitis as the starting point and followed-up for 28 days, 84 patients with severe pancreatitis were divided into survival group (62 cases) and death group (22 cases).

### Detection of serum IG % and D-D

After admission, about 5 mL of fasting venous blood was collected from all subjects. After the blood was treated, the D-D level was determined by a fully automatic hemagglutination instrument and a test kit, and the IG % level was determined by an automatic blood analyzer and its auxiliary reagents.

### Statistical methods

The data was analyzed by SPSS 24.0 statistic software. Patient's age, body mass index serum, IG % and D-D level were represented as mean ± standard deviation, and *t*- test was used to compare between groups. Classification data were reported by frequency and percent, and analyzed by Chi-Squared test. Logistic regression was used to fit the continuous variables of the multi-indicator joint assessment, and Receiver Characteristic Operating Curve (ROC) was used to evaluate the application value of IG % combined with D-D in acute pancreatitis and the severity of the disease; p < 0.05 was considered significant.

## Results

### Baseline characteristics

No visible differences in sex, age, body mass index, and basic diseases were observed between the two groups (p > 0.05) (Table 1).

**Table 1:** Clinical characteristics of patients in the two groups.

Variable	Study group (n = 84)	Control group (n = 80)	*t*/χ^2^‐values	p‐values
Sex (male/female)	36/48	38/42	0.357	0.550
Age (years)	60.74 ± 6.88	61.33 ± 6.79	0.552	0.581
Body mass index (kg/m^2^)	22.23 ± 2.08	22.56 ± 2.34	0.956	0.341
Basic diseases				
Hypertensive	15 (17.86)	7 (8.75)	3.373	0.066
Diabetes	11 (13.10)	4 (5.00)	3.145	0.076
Coronary heart disease	4 (4.76)	1 (1.25)	0.728	0.394

### The serum levels of IG % and D-D in the study and control group

The expressions of IG % and D-D in the study group were apparently higher than the control group (p < 0.05) (Table 2).

**Table 2:** Comparison of IG % and D-D between two groups.

Groups	IG %	D-D (mg/L)
Study group (n = 84)	1.35 ± 0.37	2.15 ± 0.66
Control group (n = 80)	0.97 ± 0.27	1.16 ± 0.35
*t*‐values	7.482	11.914
p‐values	0.000	0.000

### The serum levels of IG % and D-D in the survival group and death group

The expressions of IG % and D-D in the death group were distinctly higher than in the survival group (p < 0.05) (Table 3).

**Table 3:** Comparison of IG % and D-D between two groups.

Groups	IG %	D-D (mg/L)
Death group (n = 22)	1.58 ± 0.42	2.57 ± 0.68
Survival group (n = 62)	1.27 ± 0.31	2.00 ± 0.56
*t*‐values	3.657	3.873
p‐values	0.000	0.000

### Diagnostic value of IG % and D-D in severe pancreatitis

**Fig. 1:**
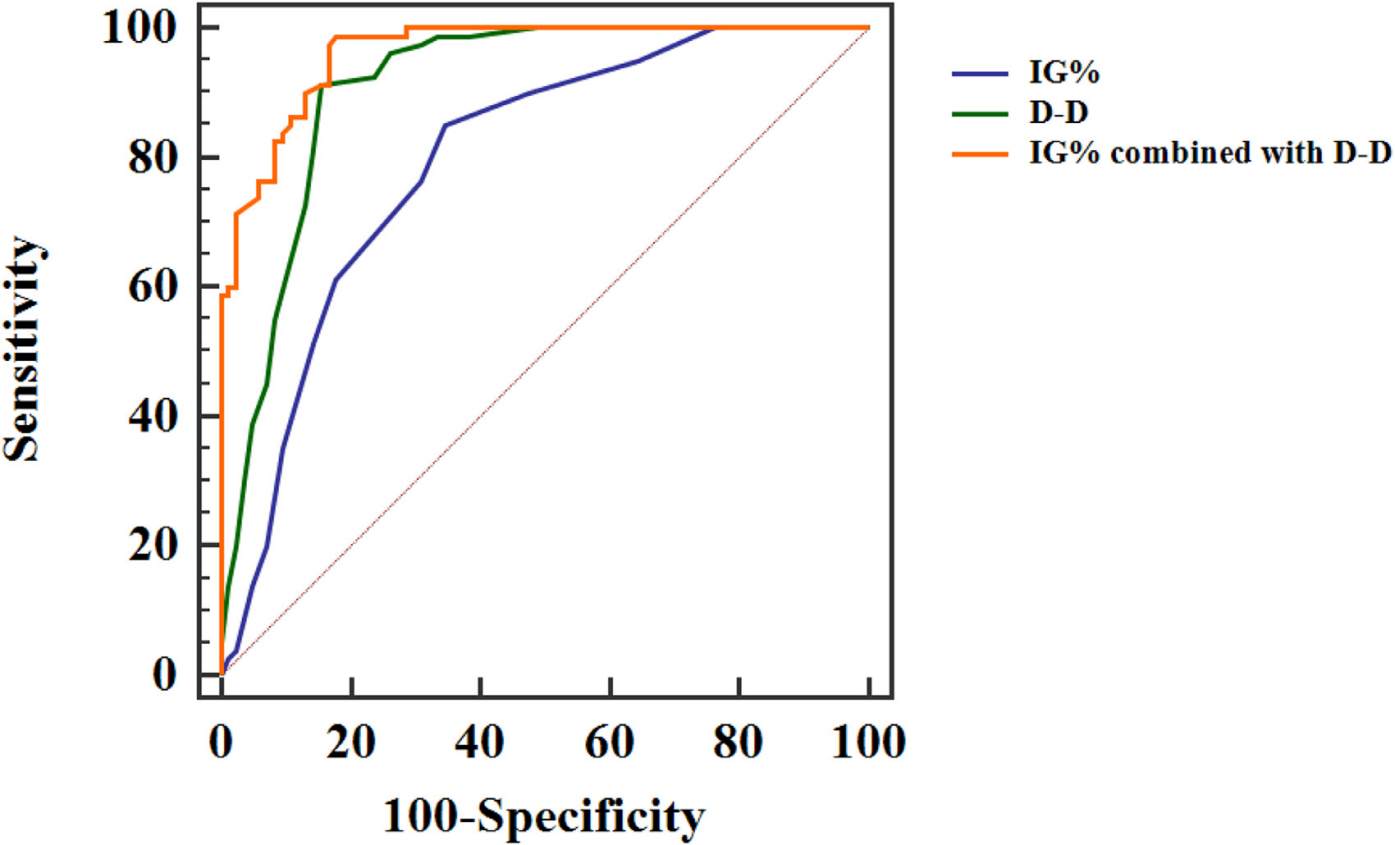
ROC curve of IG % and D-D for diagnosis of severe pancreatitis.

As can be seen from the ROC curve, the AUC of IG % and D-D combined diagnosis of severe pancreatitis was 0.963, which was significantly higher than that of individual indicator diagnosis, and the sensitivity and specificity of combined diagnosis were also significantly improved, exhibited in Fig. 1 and Table 4.

**Table 4:** Diagnostic value of IG % and D-D in severe pancreatitis.

Variables	AUC	Associated criterion	Standard error	p-values	Sensitivity	Specificity	95 % Confidence interval
IG %	0.796	1.2	0.035	<0.001	85.00	65.48	0.726∼0.854
D-D	0.810	1.5	0.024	<0.001	91.25	84.52	0.855∼0.949
IG % combined with D-D	0.963	‒	0.012	<0.001	98.75	82.14	0.921∼0.986

### Assessment value of IG % and D-D in the prognosis of severe pancreatitis patients

**Fig. 2:**
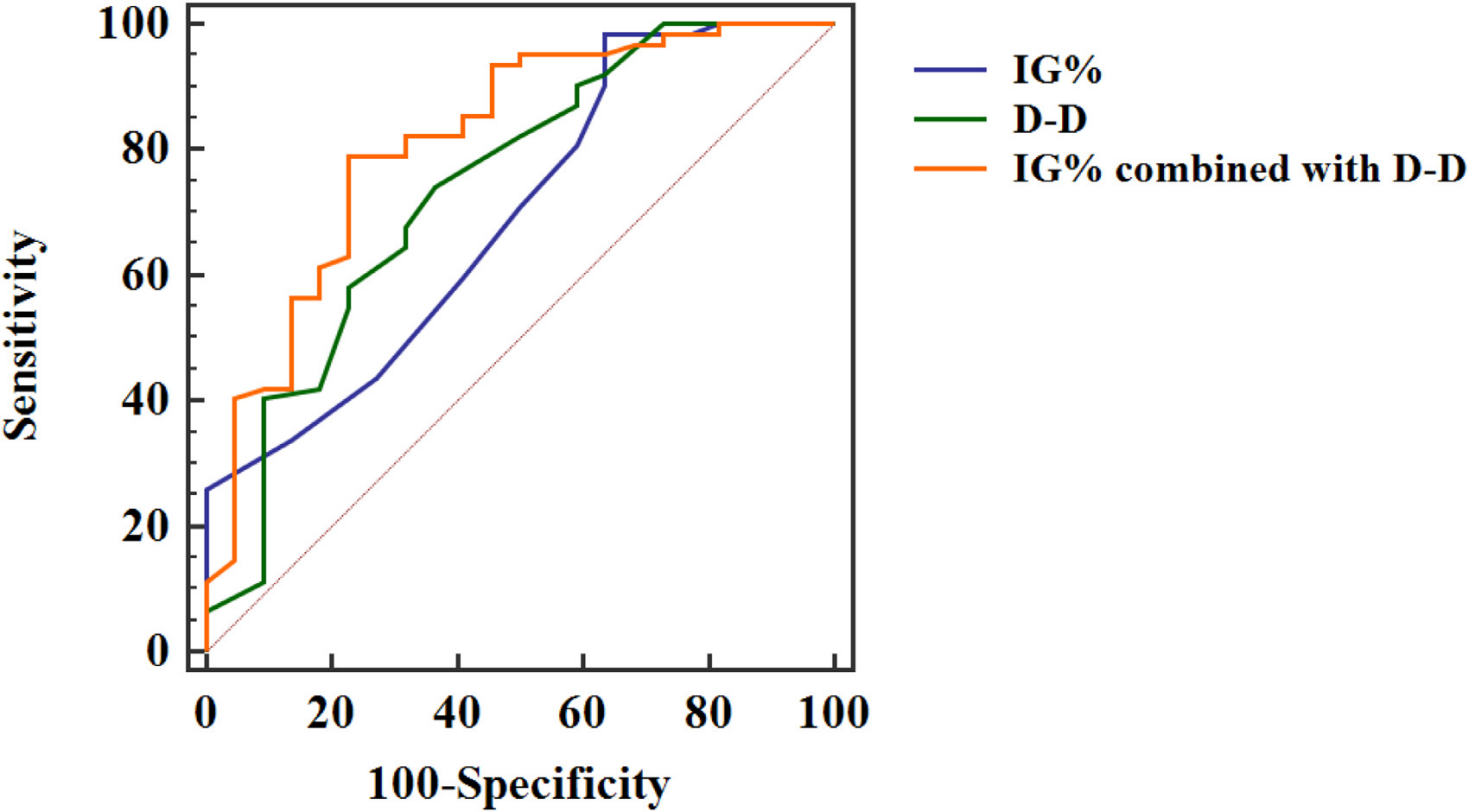
ROC curve of IG % and D-D to evaluate the prognosis of severe pancreatitis.

As can be seen from the ROC curve, the AUC of IG % and D-D combined assessment of the prognosis of patients with severe pancreatitis was 0.814, which was obviously higher than that of single indicator assessment, and the sensitivity and specificity of combined assessment were also improved observably, exhibited in Fig. 2 and Table 5.

**Table 5:** Assessment value of IG % and D-D in prognosis of severe pancreatitis patients.

Variables	AUC	Associated criterion	Standard error	p-values	Sensitivity	Specificity	95 % Confidence interval
IG %	0.692	1.7	0.035	0.004	98.39	36.36	0.582∼0.788
D-D	0.740	2.4	0.066	<0.001	74.19	63.64	0.633∼0.830
IG % combined with D-D	0.814	‒	0.056	<0.001	79.03	77.27	0.714∼0.891

## Discussion

Acute pancreatitis is an acute inflammatory response disease of the pancreas that progresses rapidly. Once acute pancreatitis develops into a severe disease, it will not only increase the difficulty of clinical treatment but also increase the death risk of patients, especially for elderly patients.^13^ Therefore, accurate and rapid assessment of the disease as early as possible can reduce the time of treatment, which has vital significance for the prognosis of patients. At present, the clinical diagnosis of severe pancreatitis is mainly based on the symptoms, signs, laboratory tests, and imaging findings of patients, but these indicators lack specificity and are easy to affect the accuracy of diagnostic results.^14^ Therefore, it is still necessary to explore more ideal indicators to improve the accuracy of diagnosis.

IG % is closely related to the occurrence and development of many diseases. In some pathological states such as infection, inflammation, and tumor, the body may experience the phenomenon of IG % increase.^15^ Currently, IG % has been regarded as a new inflammatory indicator, which has certain application value in the diagnosis and prognosis evaluation of a variety of diseases. Karakaya et al. confirmed that IG % has high sensitivity and specificity for predicting acute pyelonephritis.^16^ In Güler's study, the utility of IG % in predicting acute appendicitis was analyzed, and the results showed that IG % was a predictor for patients with moderately critical appendicitis.^17^. In Huang's research, the IG % has been proven to effectively identify Acute Respiratory Distress Syndrome (ARDS) in patients with acute pancreatitis.^18^. Bedel et al. elucidated that IG % can predict the severity of acute pancreatitis with a sensitivity of 72.7 %, which was lower than conventional clinical indicators and still needs to be enhanced.^19^ D-D is a key index reflecting the degree of fibrinolysis and has been proved to be an important reference for predicting the severity of acute pancreatitis.^20^ Therefore, this study attempted to use IG % combined with D-D to evaluate severe pancreatitis and its prognosis, in order to provide certain reference value for clinical identification of severe pancreatitis and poor prognosis.

The results of this study showed that IG % and D-D levels in the study group were higher than those in the control group, and IG % and D-D levels in the death group were higher than those in the survival group, suggesting that IG % and D-D levels can be used as indicators for the evaluation of severe pancreatitis and its prognosis. Further, ROC curve analysis showed that the AUC of IG % and D-D combined assessment of severe acute pancreatitis was 0.963, which was higher than that of IG % and D-D alone assessment. Besides, the sensitivity and specificity obtained obvious improvement. Ugurlu et al. confirmed that IG % played an important role in predicting the occurrence and development of acute pancreatitis, but it did not explore the predictive value of IG % for the prognosis of acute pancreatitis.^21^ In He's study,^22^ D-D was proved to have important predictive value for severe pancreatitis, which is similar to the results of this study. In Karakulak's study, IG % was proved to have a certain value in evaluating the prognosis of acute pancreatitis, but its specificity was only 50.00 %, suggesting the necessity of IG % combined with other indicators.^23^ In this research, the value of IG % combined with D-D in evaluating the prognosis of severe pancreatitis was further explored. The results showed the AUC of IG % and D-D combined evaluation for the prognosis of severe pancreatitis was 0.814, and the sensitivity and specificity were 79.03 % and 77.27 %, respectively, which was significantly improved than that of each index alone. These results further illustrate that IG %, and D-D can be the indicators for the evaluation of severe pancreatitis and its prognosis, while the combination of both indicators can effectively improve the evaluation efficiency.

## Conclusion

In conclusion, serum IG % and D-D levels are highly expressed in patients with severe pancreatitis, so the detection of IG % and D-D levels can provide an important reference value for the evaluation of severe pancreatitis and its prognosis. In addition, the combination of IG % and D-D can improve the evaluation efficacy of severe pancreatitis and its prognosis.

Despite its findings, this study is not without flaws. This study is a single-center retrospective analysis, not a large sample study. In the future, multi-center and large sample size prospective research are still needed to verify and promote the results of this study, so as to provide a more scientific basis for the early diagnosis and treatment of patients with severe acute pancreatitis, further improving the prognosis of patients.

## Ethics approval and consent to participate

The ethics approval was reviewed and approved by The Shengjing Hospital, China.

Medical University and written informed consent was obtained from all patients. The ethical approval number was 2020062. The study follows the STROBE Statement.

## Consent for publishing

All of the authors have consented to publish this research.

## Funding

This research received no external funding.

## Conflicts of interest

The authors declare no conflicts of interest.

## CRediT authorship contribution statement

**Tian-Tian Xu:** Conceptualization, Methodology, Software, Validation, Formal analysis, Investigation, Resources, Data curation, Writing – original draft, Writing – review & editing, Visualization, Supervision, Project administration. **Si-Bing Chen:** Conceptualization, Methodology, Software, Validation, Formal analysis, Resources, Writing – review & editing, Visualization, Supervision, Project administration, Funding acquisition.
